# Supplementary material for: Rapid and Visual Detection of Volatile Amines Based on Their Gas–Solid Reaction with Tetrachloro-p-Benzoquinone
Source: Molecules. 2024 Apr 17;29(8):1818. doi: 10.3390/molecules29081818 (PMC11054329; doi:10.3390/molecules29081818)

checkCIF/PLATON report

You have not supplied any structure factors. As a result the full set of tests cannot be run.

THIS REPORT IS FOR GUIDANCE ONLY. IF USED AS PART OF A REVIEW PROCEDURE FOR PUBLICATION, IT SHOULD NOT REPLACE THE EXPERTISE OF AN EXPERIENCED CRYSTALLOGRAPHIC REFEREE.

No syntax errors found.   CIF dictionary   Interpreting this report

Datablock: 2403145_0m

```
Bond precision:   C-C = 0.0075 A           Wavelength=0.71073

Cell:             a=7.426(6)     b=4.668(4)           c=22.114(19)
                  alpha=90       beta=93.448(11)      gamma=90
Temperature:      296 K

                 Calculated                  Reported
Volume           765.2(11)                   765.1(12)
Space group      P 21/n                      P 1 21/n 1
Hall group       -P 2yn                      -P 2yn
Moiety formula   C14 H20 Cl2 N2 O2           C14 H20 Cl2 N2 O2
Sum formula      C14 H20 Cl2 N2 O2           C14 H20 Cl2 N2 O2
Mr               319.22                      319.22
Dx,g cm-3        1.386                       1.386
Z                2                           2
Mu (mm-1)        0.427                       0.427
F000             336.0                       336.0
F000'            336.71
h,k,lmax         8,5,26                      8,5,26
Nref             1355                        1302
Tmin,Tmax        0.825,0.914                 0.493,0.746
Tmin'            0.825

Correction method= # Reported T Limits: Tmin=0.493 Tmax=0.746
AbsCorr = MULTI-SCAN

Data completeness= 0.961          Theta(max)= 24.978

R(reflections)= 0.0788( 780)                          wR2(reflections)=
                                                      0.1639( 1302)
S = 1.157                   Npar= 92
```

The following ALERTS were generated. Each ALERT has the format
test-name_ALERT_alert-type_alert-level.
Click on the hyperlinks for more details of the test.

Alert level C

```
PLAT029_ALERT_3_C _diffrn_measured_fraction_theta_full value Low .      0.961 Why?
PLAT340_ALERT_3_C Low Bond Precision on  C-C Bonds ...............     0.0075 Ang.
```

Alert level G

```
PLAT007_ALERT_5_G Number of Unrefined Donor-H Atoms .............          1 Report
              H003
PLAT333_ALERT_2_G Large Aver C6-Ring C-C Dist C004     -C006_a   .       1.42 Ang.
PLAT335_ALERT_2_G Check Large C6 Ring C-C Range C004     -C006_a         0.16 Ang.
PLAT720_ALERT_4_G Number of Unusual/Non-Standard Labels ..........         20 Note
              Cl01    O002    N003    H003    C004    C005    C006    C007
              H00A    H00B    C008    H00C    H00D    C009    H00E    H00F
              C00A    H00G    H00H    H00I
PLAT933_ALERT_2_G Number of HKL-OMIT Records in Embedded .res File          4 Note
               -8  1  8,  -8  1 11,  -7  0 15,  -7  0 17,
PLAT941_ALERT_3_G Average HKL Measurement Multiplicity ...........        3.9 Low
PLAT967_ALERT_5_G Note: Two-Theta Cutoff Value in Embedded .res ..       50.0 Degree
```

0 **ALERT level A** = Most likely a serious problem - resolve or explain
0 **ALERT level B** = A potentially serious problem, consider carefully
2 **ALERT level C** = Check. Ensure it is not caused by an omission or oversight
7 **ALERT level G** = General information/check it is not something unexpected

0 ALERT type 1 CIF construction/syntax error, inconsistent or missing data
3 ALERT type 2 Indicator that the structure model may be wrong or deficient
3 ALERT type 3 Indicator that the structure quality may be low
1 ALERT type 4 Improvement, methodology, query or suggestion
2 ALERT type 5 Informative message, check

It is advisable to attempt to resolve as many as possible of the alerts in all categories. Often the minor alerts point to easily fixed oversights, errors and omissions in your CIF or refinement strategy, so attention to these fine details can be worthwhile. In order to resolve some of the more serious problems it may be necessary to carry out additional measurements or structure refinements. However, the purpose of your study may justify the reported deviations and the more serious of these should normally be commented upon in the discussion or experimental section of a paper or in the "special_details" fields of the CIF. checkCIF was carefully designed to identify outliers and unusual parameters, but every test has its limitations and alerts that are not important in a particular case may appear. Conversely, the absence of alerts does not guarantee there are no aspects of the results needing attention. It is up to the individual to critically assess their own results and, if necessary, seek expert advice.

Publication of your CIF in IUCr journals

A basic structural check has been run on your CIF. These basic checks will be run on all CIFs submitted for publication in IUCr journals (*Acta Crystallographica*, *Journal of Applied Crystallography*, *Journal of Synchrotron Radiation*); however, if you intend to submit to *Acta Crystallographica Section C* or *E* or *IUCrData*, you should make sure that full publication checks are run on the final version of your CIF prior to submission.

Publication of your CIF in other journals

Please refer to the *Notes for Authors* of the relevant journal for any special instructions relating to CIF submission.

PLATON version of 06/01/2024; check.def file version of 05/01/2024

Datablock 2403145_0m - ellipsoid plot

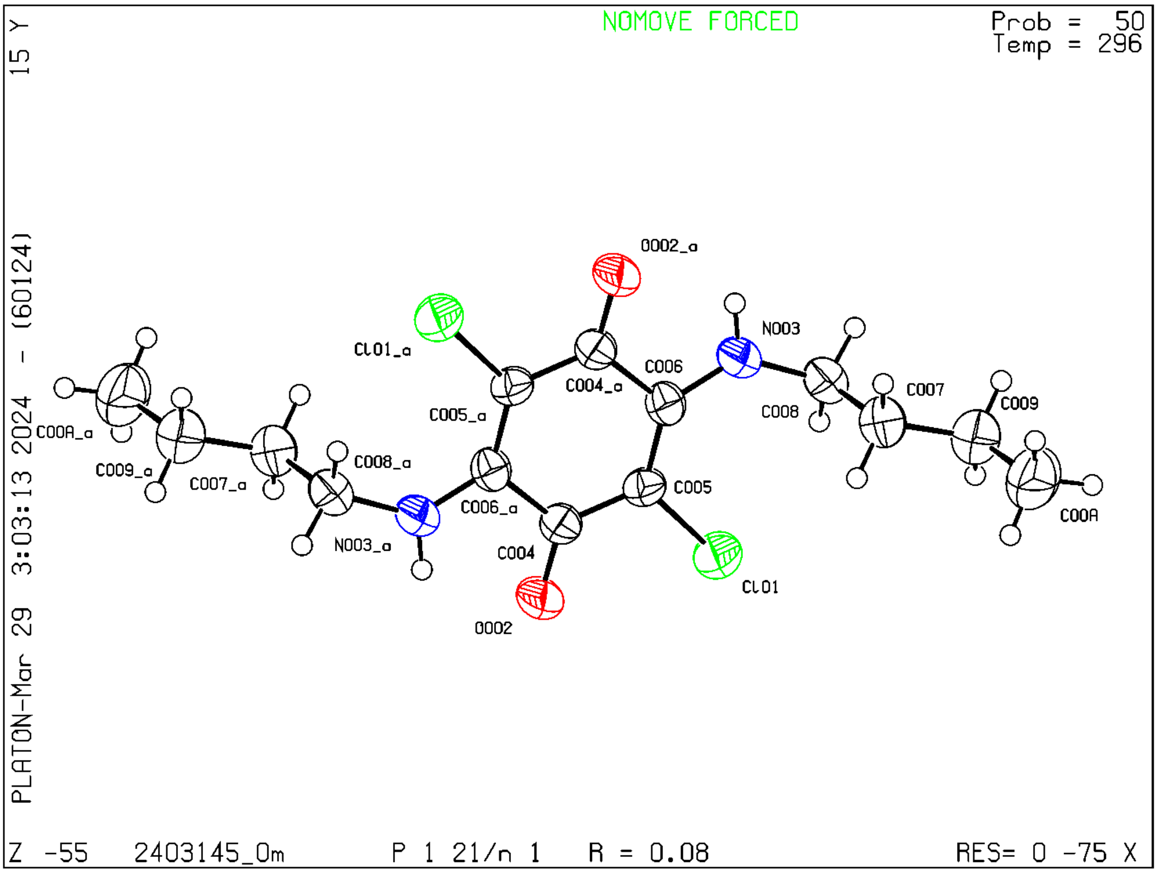

Supplement: Supplementary file 1 [file molecules-29-01818-s001.zip › crystal data/checkcif C4.pdf]
