# Supplementary material for: Rapid and Visual Detection of Volatile Amines Based on Their Gas–Solid Reaction with Tetrachloro-p-Benzoquinone
Source: Molecules. 2024 Apr 17;29(8):1818. doi: 10.3390/molecules29081818 (PMC11054329; doi:10.3390/molecules29081818)

checkCIF/PLATON report

Structure factors have been supplied for datablock(s) 66_a

THIS REPORT IS FOR GUIDANCE ONLY. IF USED AS PART OF A REVIEW PROCEDURE FOR PUBLICATION, IT SHOULD NOT REPLACE THE EXPERTISE OF AN EXPERIENCED CRYSTALLOGRAPHIC REFEREE.

No syntax errors found. CIF dictionary Interpreting this report

Datablock: 66_a

Bond precision: C-C = 0.0110 A Wavelength=0.71073

Cell:	a=4.722(15)	b=7.40(2)	c=14.97(5)
	alpha=91.92(4)	beta=97.45(3)	gamma=107.18(3)
Temperature:	296 K		

	Calculated	Reported
Volume	494(3)	494(3)
Space group	P -1	P -1
Hall group	-P 1	-P 1
Moiety formula	C18 H28 Cl2 N2 O2	C18 H28 Cl2 N2 O2
Sum formula	C18 H28 Cl2 N2 O2	C18 H28 Cl2 N2 O2
Mr	375.32	375.32
Dx,g cm-3	1.262	1.262
Z	1	1
Mu (mm-1)	0.341	0.341
F000	200.0	200.0
F000’	200.36	
h,k,lmax	5,8,17	5,8,17
Nref	1734	1712
Tmin,Tmax	0.934,0.966	0.632,0.746
Tmin’	0.934	

Correction method= # Reported T Limits: Tmin=0.632 Tmax=0.746
AbsCorr = MULTI-SCAN

Data completeness= 0.987 Theta(max)= 24.999

R(reflections)= 0.0817(1313) wR2(reflections)= 0.2477(1712)

S = 1.127 Npar= 110

The following ALERTS were generated. Each ALERT has the format
test-name_ALERT_alert-type_alert-level.
Click on the hyperlinks for more details of the test.

Alert level B

```
PLAT340_ALERT_3_B Low Bond Precision on  C-C Bonds ..............      0.011 Ang.
```

Alert level C

```
PLAT148_ALERT_3_C s.u. on the      a    - Axis is (Too) Large ....      0.015 Ang.
PLAT148_ALERT_3_C s.u. on the      b    - Axis is (Too) Large ....      0.020 Ang.
PLAT148_ALERT_3_C s.u. on the      c    - Axis is (Too) Large ....      0.050 Ang.
PLAT220_ALERT_2_C NonSolvent   Resd 1  C   Ueq(max)/Ueq(min) Range        3.6 Ratio
PLAT234_ALERT_4_C Large Hirshfeld Difference C5       --C6       .       0.16 Ang.
PLAT234_ALERT_4_C Large Hirshfeld Difference C7       --C8       .       0.16 Ang.
PLAT906_ALERT_3_C Large K Value in the Analysis of Variance ......      8.221 Check
PLAT906_ALERT_3_C Large K Value in the Analysis of Variance ......      2.023 Check
PLAT911_ALERT_3_C Missing FCF Refl Between Thmin & STh/L=    0.595         22 Report
                0  4  0,  -4  7  0,   0 -3  1,   3 -8  2,  -1 -4  2,  -4 -3  2,
                0  3  2,   3 -8  3,  -4 -4  3,   1 -4  3,   4 -7  4,   4 -6  4,
                4 -5  4,  -4 -4  4,  -2  2  4,   3 -8  5,   4 -7  5,   4 -6  5,
                4 -5  5,   3  2  7,  -2  4  7,  -1  2 17,
```

Alert level G

```
PLAT002_ALERT_2_G Number of Distance or Angle Restraints on AtSite          6 Note
PLAT003_ALERT_2_G Number of Uiso or Uij Restrained non-H Atoms ...          2 Report
PLAT007_ALERT_5_G Number of Unrefined Donor-H Atoms .............          1 Report
              H1
PLAT072_ALERT_2_G SHELXL First  Parameter in WGHT  Unusually Large       0.11 Report
PLAT172_ALERT_4_G The CIF-Embedded .res File Contains DFIX Records          1 Report
PLAT186_ALERT_4_G The CIF-Embedded .res File Contains ISOR Records          1 Report
PLAT333_ALERT_2_G Large Aver C6-Ring C-C Dist C1       -C3_a      .       1.43 Ang.
PLAT860_ALERT_3_G Number of Least-Squares Restraints ............         16 Note
PLAT909_ALERT_3_G Percentage of I>2sig(I) Data at Theta(Max) Still        44% Note
PLAT930_ALERT_2_G FCF-based   Twin Law ( 0 0 1)         Est.d BASF       0.09 Check
PLAT931_ALERT_5_G CIFcalcFCF  Twin Law ( 0 0 1)         Est.d BASF       0.09 Check
PLAT941_ALERT_3_G Average HKL Measurement Multiplicity ..........        1.9 Low
PLAT967_ALERT_5_G Note: Two-Theta Cutoff Value in Embedded .res ..       50.0 Degree
PLAT969_ALERT_5_G The 'Henn et al.' R-Factor-gap value ..........       4.57 Note
              Predicted wR2: Based on SigI**2  5.42 or SHELX Weight 22.73
PLAT978_ALERT_2_G Number C-C Bonds with Positive Residual Density.          1 Info
```

0 **ALERT level A** = Most likely a serious problem - resolve or explain
1 **ALERT level B** = A potentially serious problem, consider carefully
9 **ALERT level C** = Check. Ensure it is not caused by an omission or oversight
15 **ALERT level G** = General information/check it is not something unexpected

0 ALERT type 1 CIF construction/syntax error, inconsistent or missing data
7 ALERT type 2 Indicator that the structure model may be wrong or deficient
10 ALERT type 3 Indicator that the structure quality may be low
4 ALERT type 4 Improvement, methodology, query or suggestion
4 ALERT type 5 Informative message, check

Validation response form

Please find below a validation response form (VRF) that can be filled in and pasted into your CIF.

```
# start Validation Reply Form
_vrf_PLAT340_66_a
;
PROBLEM: Low Bond Precision on  C-C Bonds ..............      0.011 Ang.
RESPONSE: ...
;
# end Validation Reply Form
```

It is advisable to attempt to resolve as many as possible of the alerts in all categories. Often the minor alerts point to easily fixed oversights, errors and omissions in your CIF or refinement strategy, so attention to these fine details can be worthwhile. In order to resolve some of the more serious problems it may be necessary to carry out additional measurements or structure refinements. However, the purpose of your study may justify the reported deviations and the more serious of these should normally be commented upon in the discussion or experimental section of a paper or in the "special_details" fields of the CIF. checkCIF was carefully designed to identify outliers and unusual parameters, but every test has its limitations and alerts that are not important in a particular case may appear. Conversely, the absence of alerts does not guarantee there are no aspects of the results needing attention. It is up to the individual to critically assess their own results and, if necessary, seek expert advice.

Publication of your CIF in IUCr journals

A basic structural check has been run on your CIF. These basic checks will be run on all CIFs submitted for publication in IUCr journals (*Acta Crystallographica*, *Journal of Applied Crystallography*, *Journal of Synchrotron Radiation*); however, if you intend to submit to *Acta Crystallographica Section C* or *E* or *IUCrData*, you should make sure that full publication checks are run on the final version of your CIF prior to submission.

Publication of your CIF in other journals

Please refer to the *Notes for Authors* of the relevant journal for any special instructions relating to CIF submission.

PLATON version of 06/01/2024; check.def file version of 05/01/2024

Datablock 66_a - ellipsoid plot

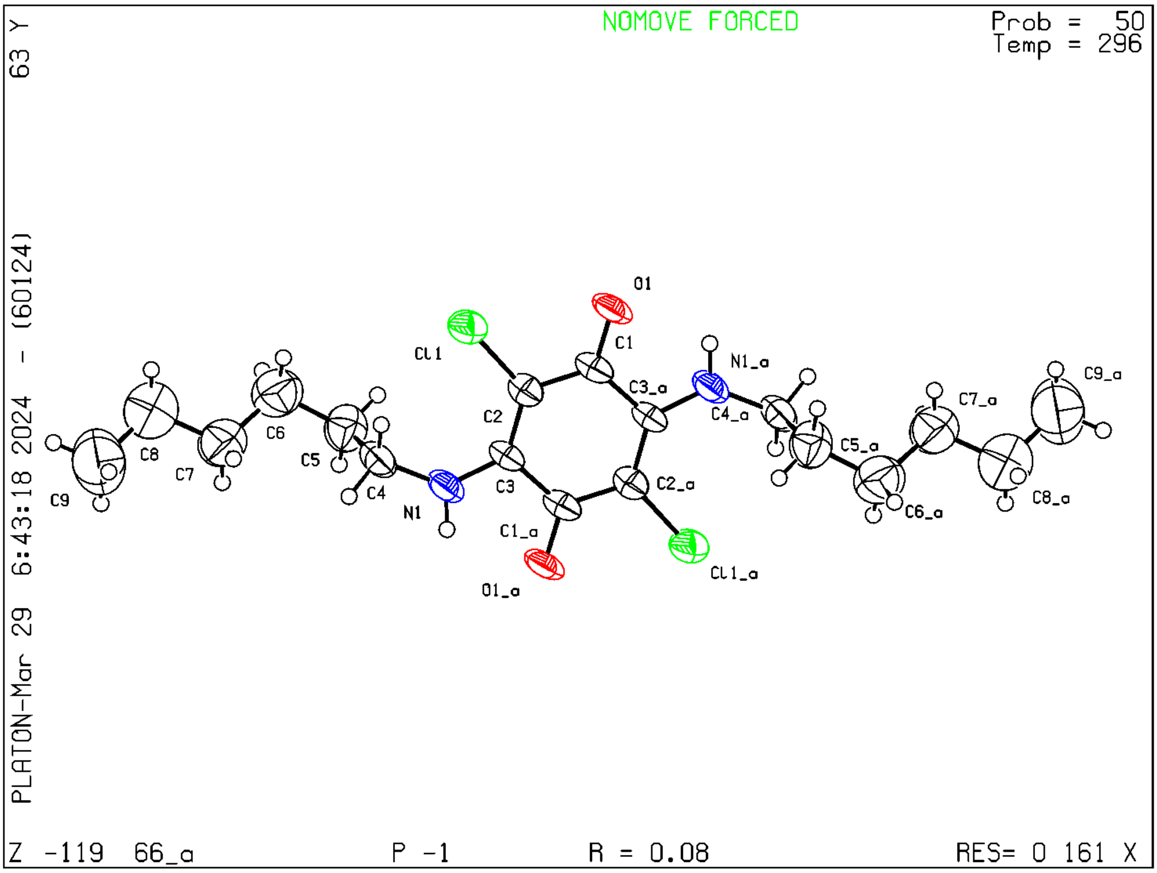

Supplement: Supplementary file 1 [file molecules-29-01818-s001.zip › crystal data/checkcif C6.pdf]
