# Supplementary material for: Rapid and Visual Detection of Volatile Amines Based on Their Gas–Solid Reaction with Tetrachloro-p-Benzoquinone
Source: Molecules. 2024 Apr 17;29(8):1818. doi: 10.3390/molecules29081818 (PMC11054329; doi:10.3390/molecules29081818)
Supplement: Supplementary file 1 [file molecules-29-01818-s001.zip › Supporting information.pdf]

Supporting Information

Rapid and Visual Detection of Volatile Amines Based on Their Gas–Solid Reaction with Tetrachloro-p-Benzoquinone

Yue-Xiang Sun, Zi-Jian Yan, Wan-Xia Liu, Xiao-Ming Chen, Man-Hua Ding, Lin-Li Tang * and Fei Zeng *

Contents

1. ^{1}H NMR and ^{13}C NMR Spectral of disubstituted products of TCBQ. 3

2. Photos of TCBQ before and after exposure to different vapor amines. ...9

3. Crystal data 10

4. Videos of TCBQ color change caused by n-propylamine 14

1. ^{1}H NMR and ^{13}C NMR Spectral of disubstituted products of TCBQ.

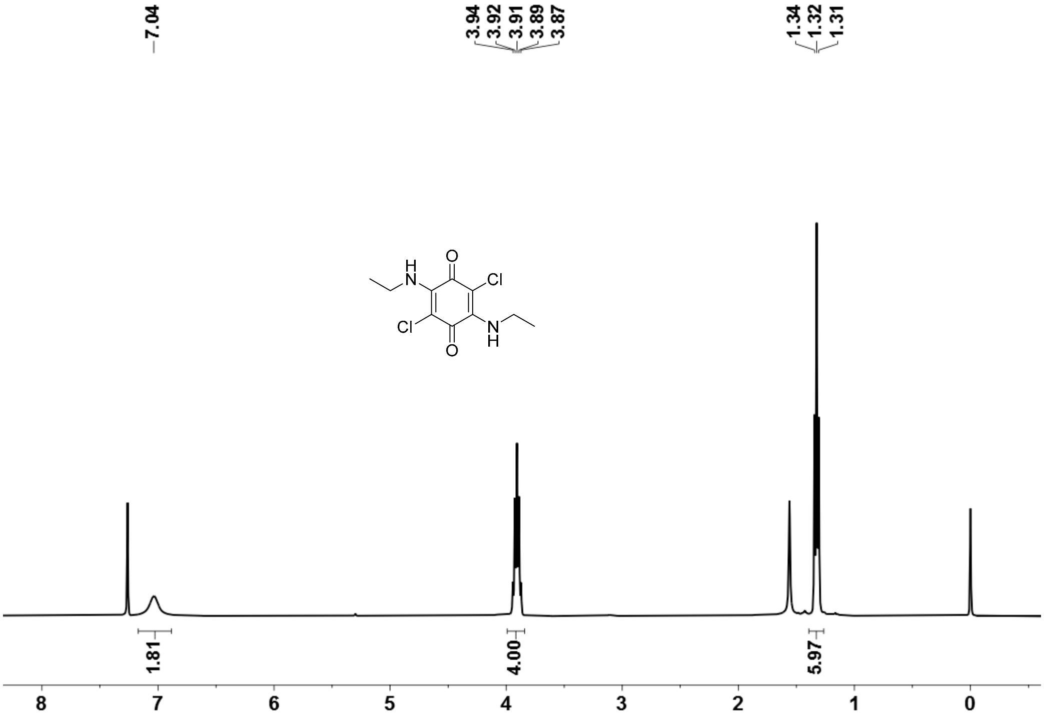

Figure S1.^{1}H NMR spectrum (400 MHz, $CDCl_3$, 298K) of 2,5-dichloro-3,6-bis(ethylamino)cyclohexa-2,5-diene-1,4-dione

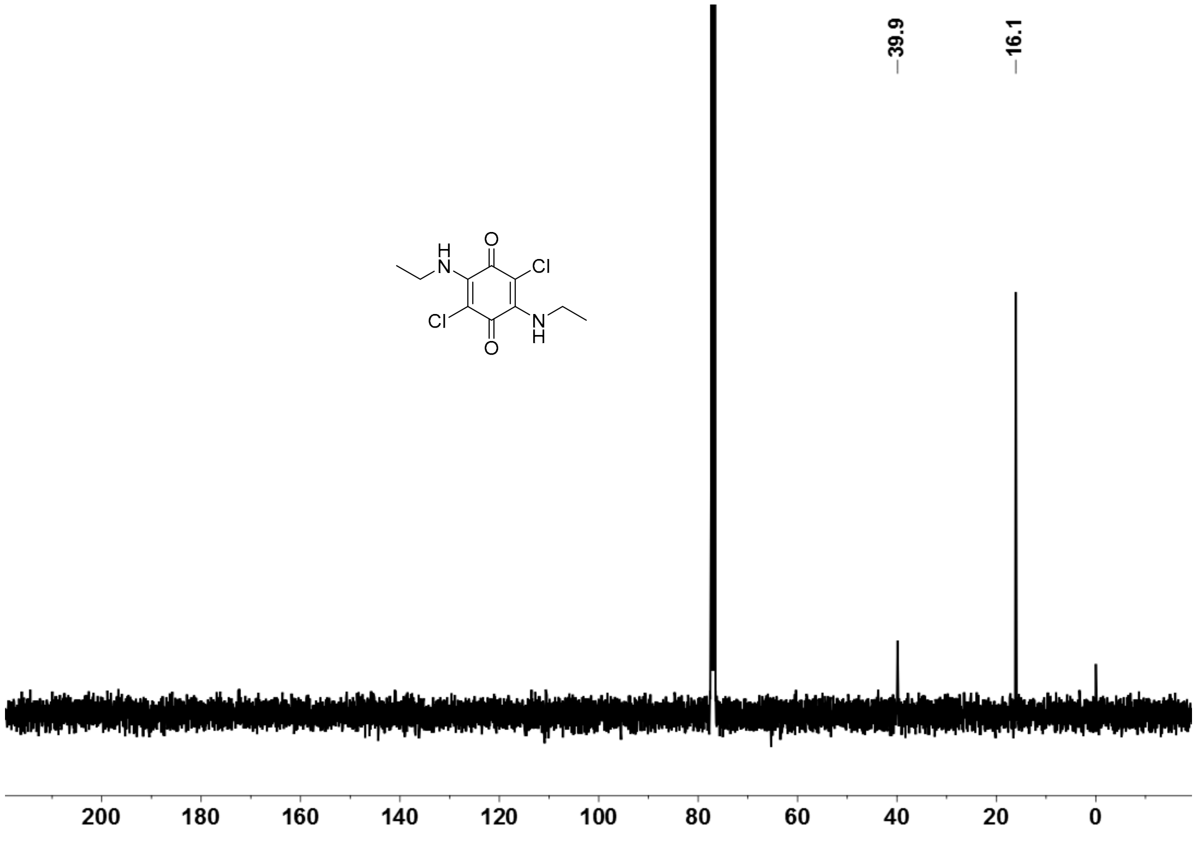

Figure S2.^{13}C NMR spectrum (101 MHz, $CDCl_3$, 298K) of 2,5-dichloro-3,6-bis(ethylamino)cyclohexa-2,5-diene-1,4-dione

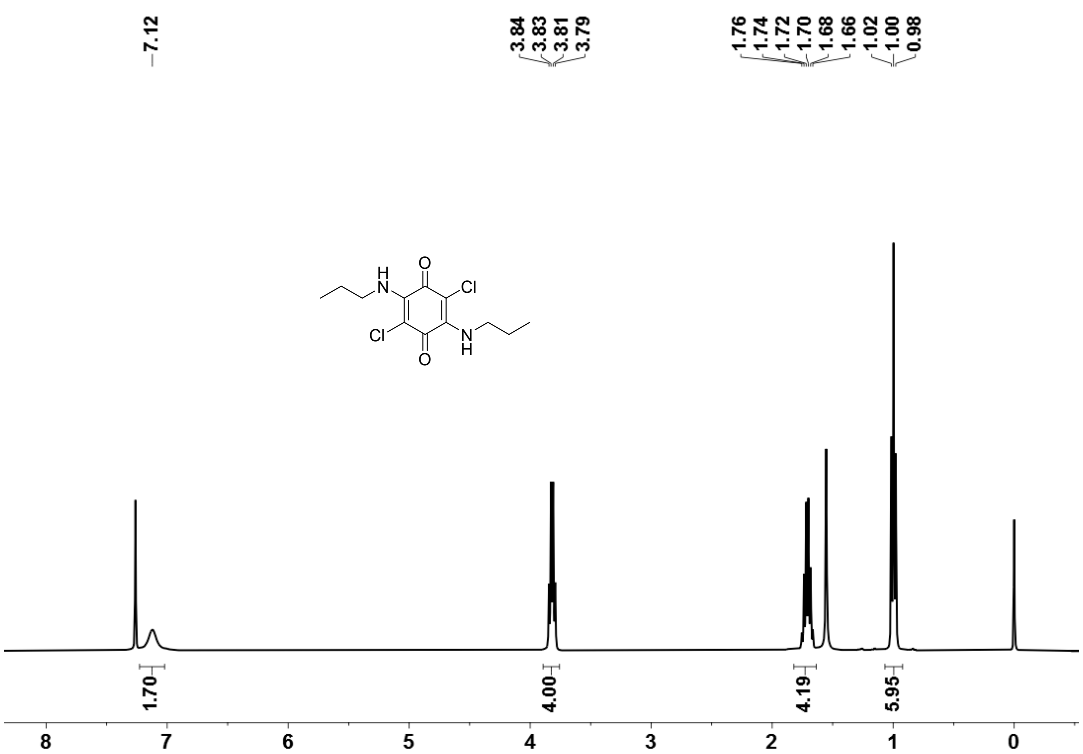

Figure S3.^{1}H NMR spectrum (400 MHz, $CDCl_3$, 298K) of 2,5-dichloro-3,6-bis(propylamino)cyclohexa-2,5-diene-1,4-dione

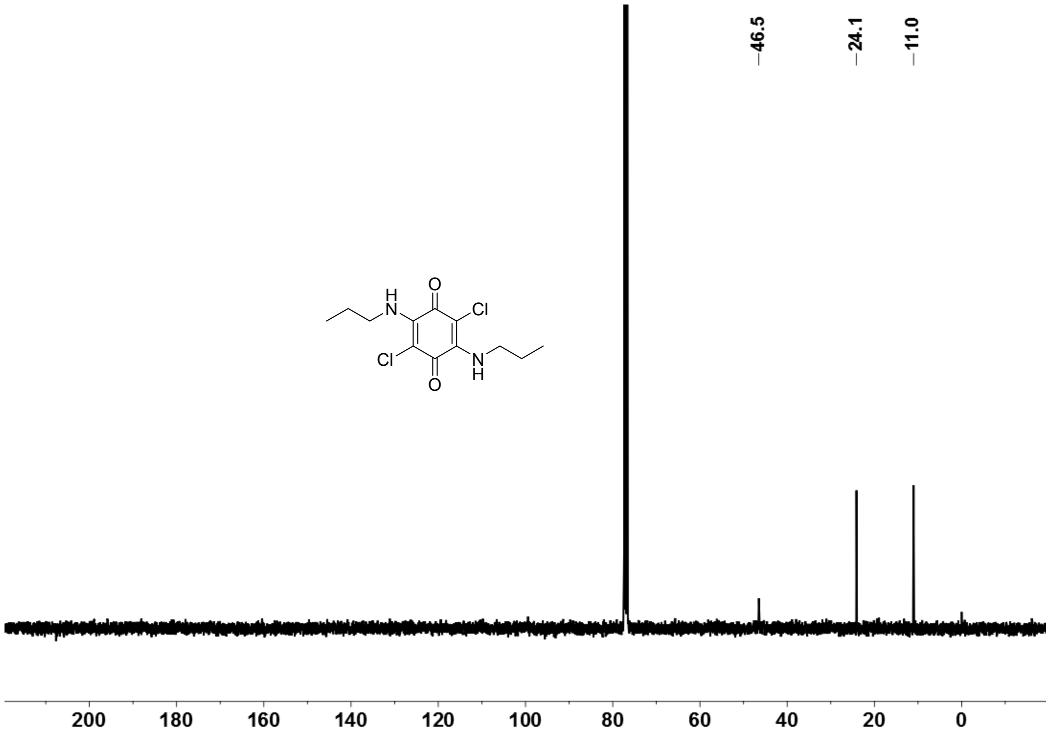

Figure S4.^{13}C NMR spectrum (101 MHz, $CDCl_3$, 298K) of 2,5-dichloro-3,6-bis(propylamino)cyclohexa-2,5-diene-1,4-dione

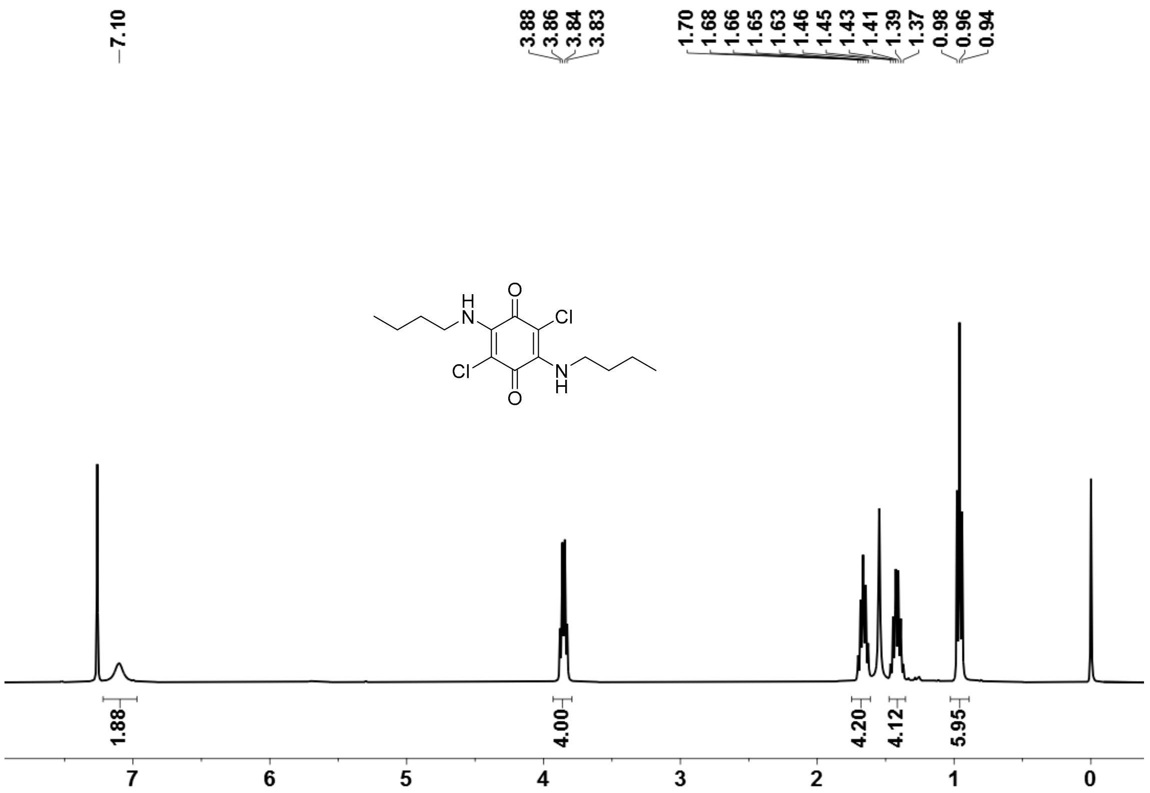

Figure S5.^{1}H NMR spectrum (400 MHz, $CDCl_3$, 298K) of 2,5-bis(butylamino)-3,6-dichlorocyclohexa-2,5-diene-1,4-dione

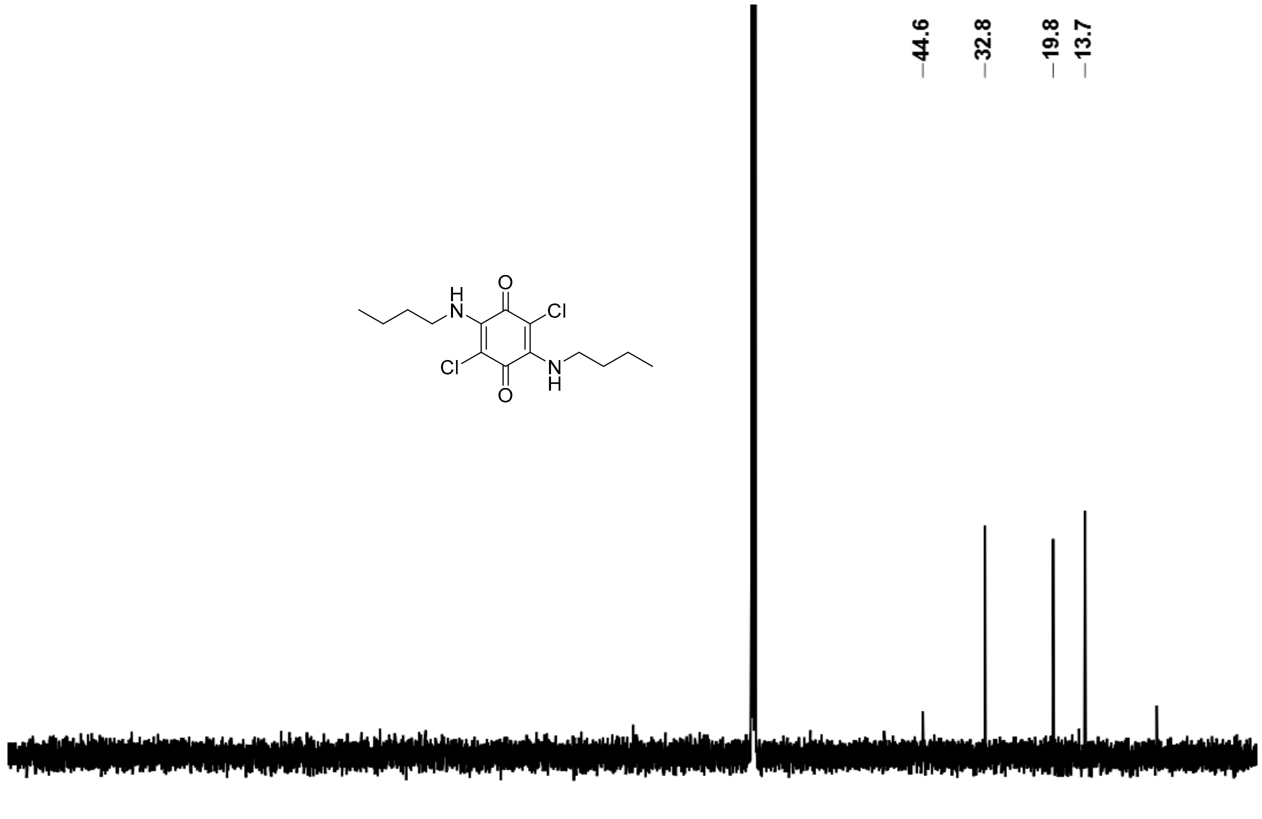

Figure S6.^{13}C NMR spectrum (101 MHz, $CDCl_3$, 298K) of 2,5-bis(butylamino)-3,6-dichlorocyclohexa-2,5-diene-1,4-dione

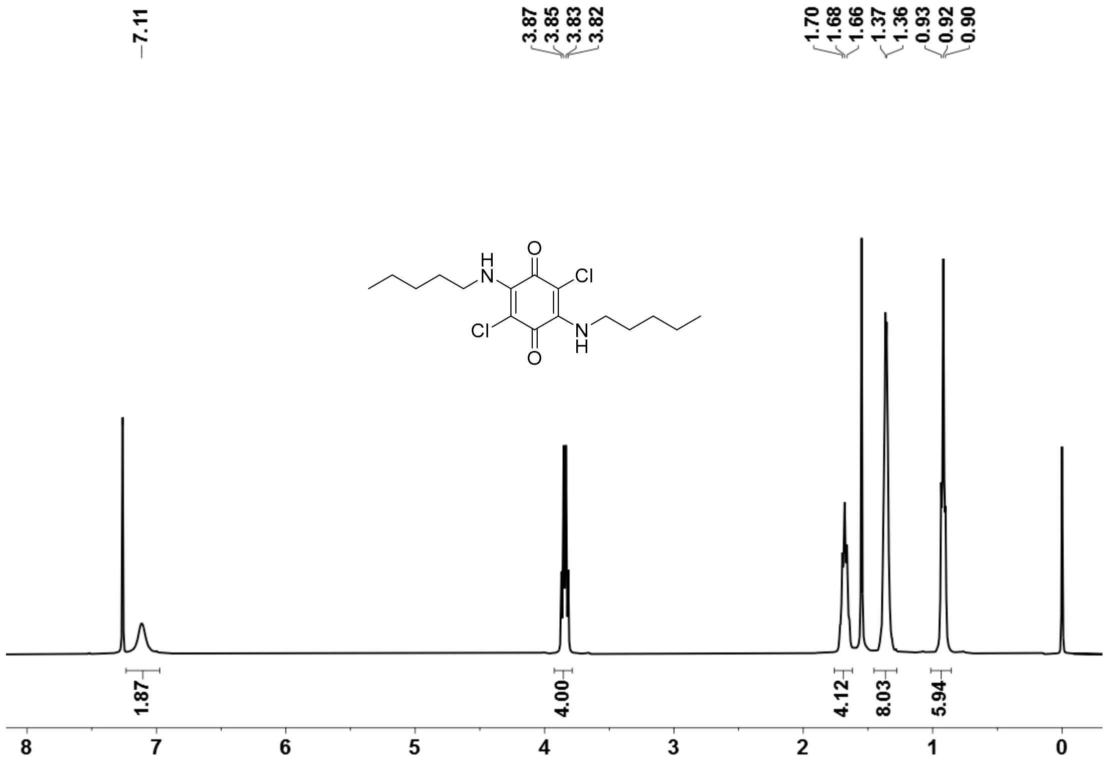

Figure S7.^{1}H NMR spectrum (400 MHz, $CDCl_3$, 298K) of 2,5-dichloro-3,6-bis(pentylamino)cyclohexa-2,5-diene-1,4-dione

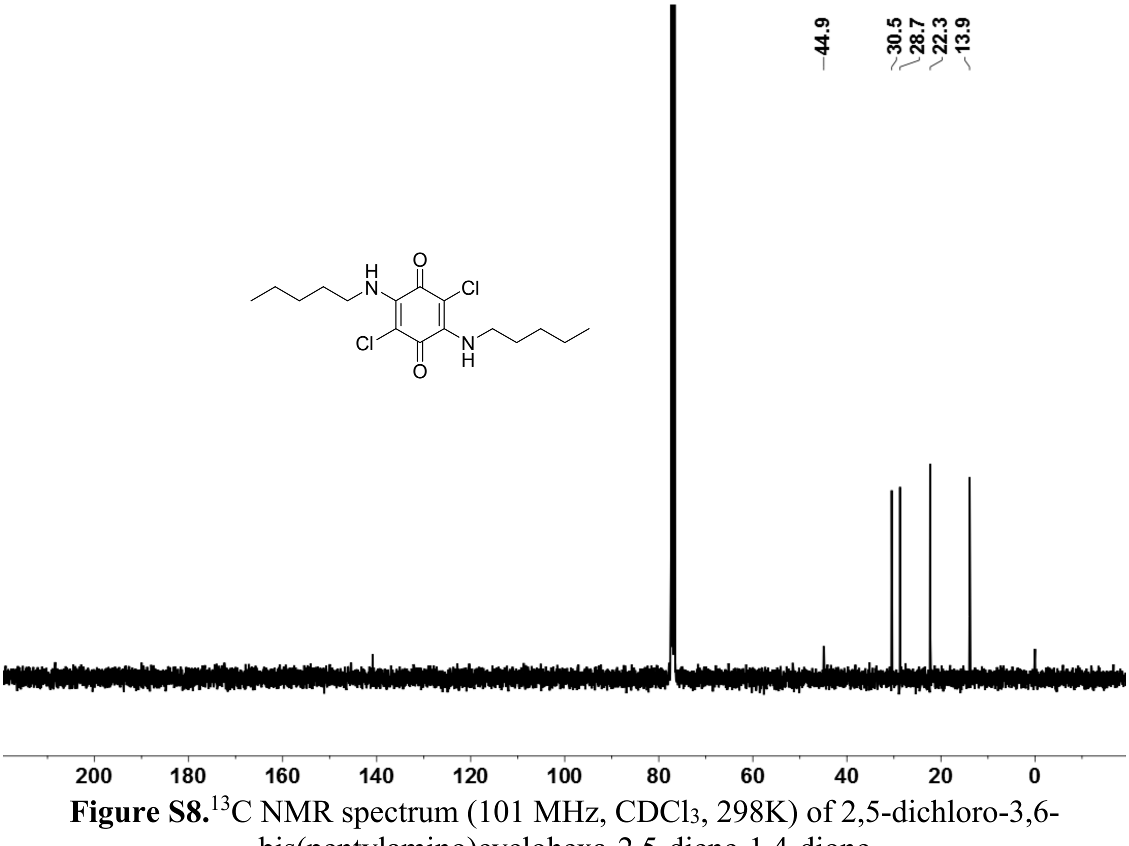

Figure S8.^{13}C NMR spectrum (101 MHz, $CDCl_3$, 298K) of 2,5-dichloro-3,6-bis(pentylamino)cyclohexa-2,5-diene-1,4-dione

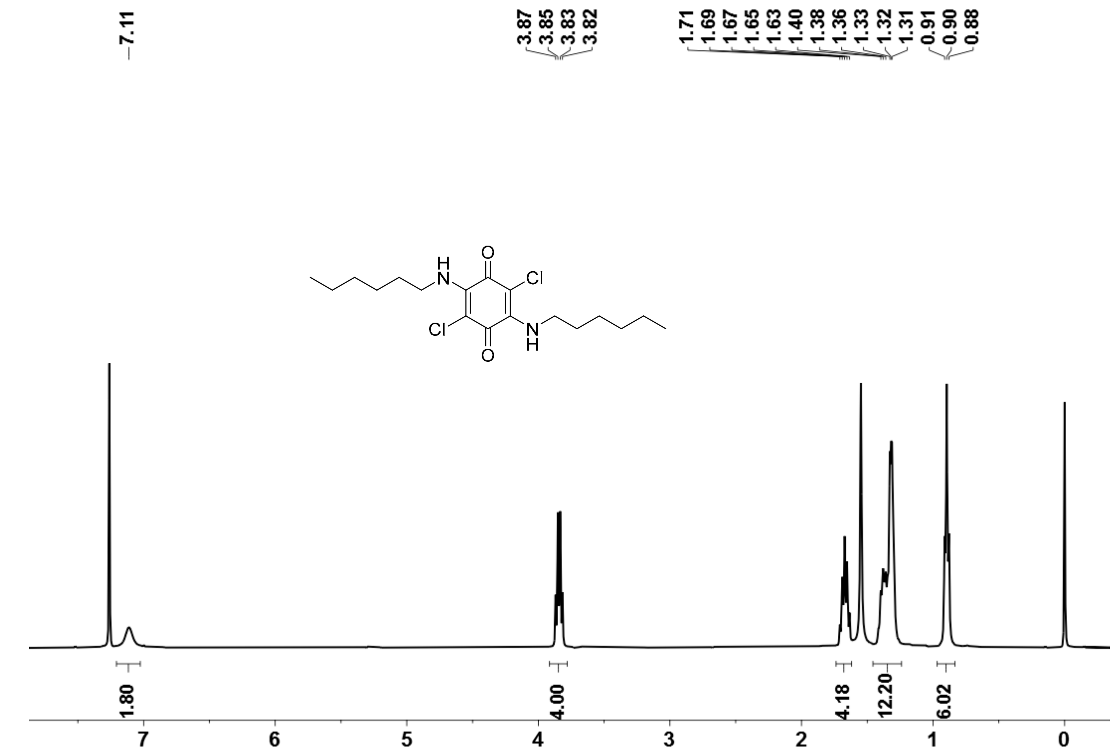

Figure S9. ^{1}H NMR spectrum (400 MHz, $CDCl_3$, 298K) of 2,5-dichloro-3,6-bis(hexylamino)cyclohexa-2,5-diene-1,4-dione

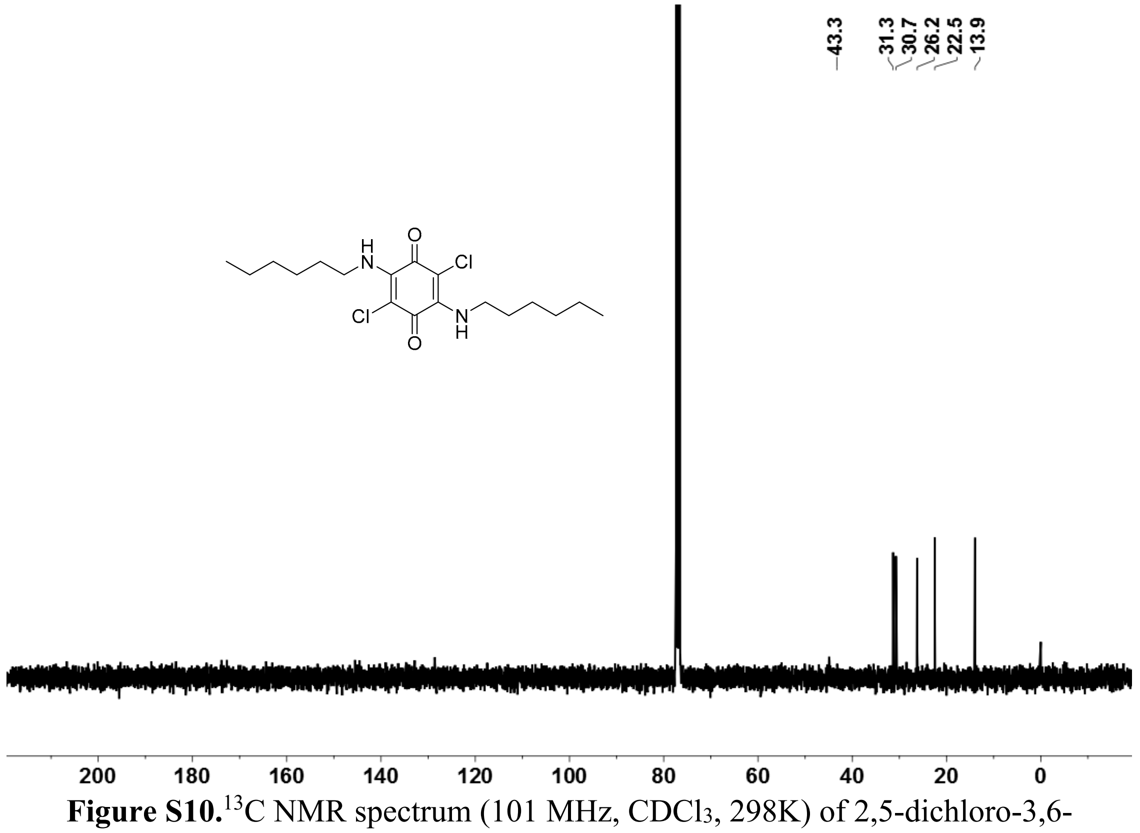

Figure S10. ^{13}C NMR spectrum (101 MHz, $CDCl_3$, 298K) of 2,5-dichloro-3,6-bis(hexylamino)cyclohexa-2,5-diene-1,4-dione

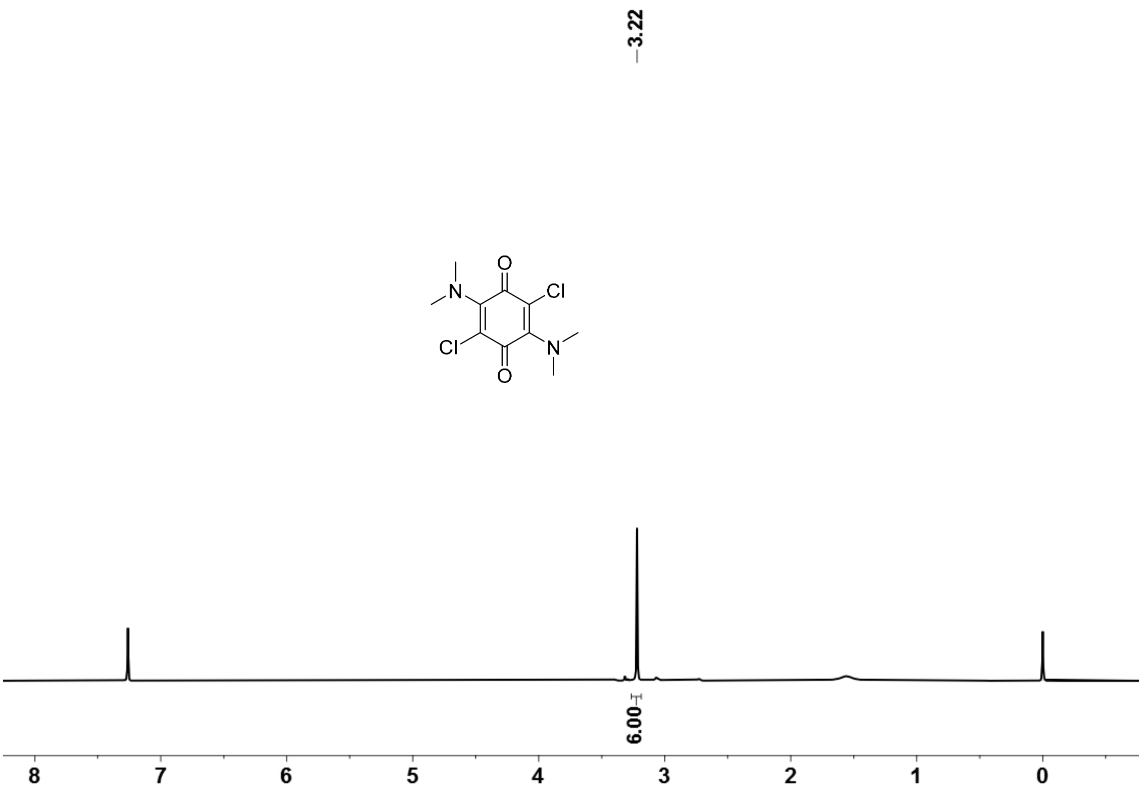

Figure S11.^{1}H NMR spectrum (400 MHz, $CDCl_3$, 298K) of 2,5-dichloro-3,6-bis(dimethylamino)cyclohexa-2,5-diene-1,4-dione

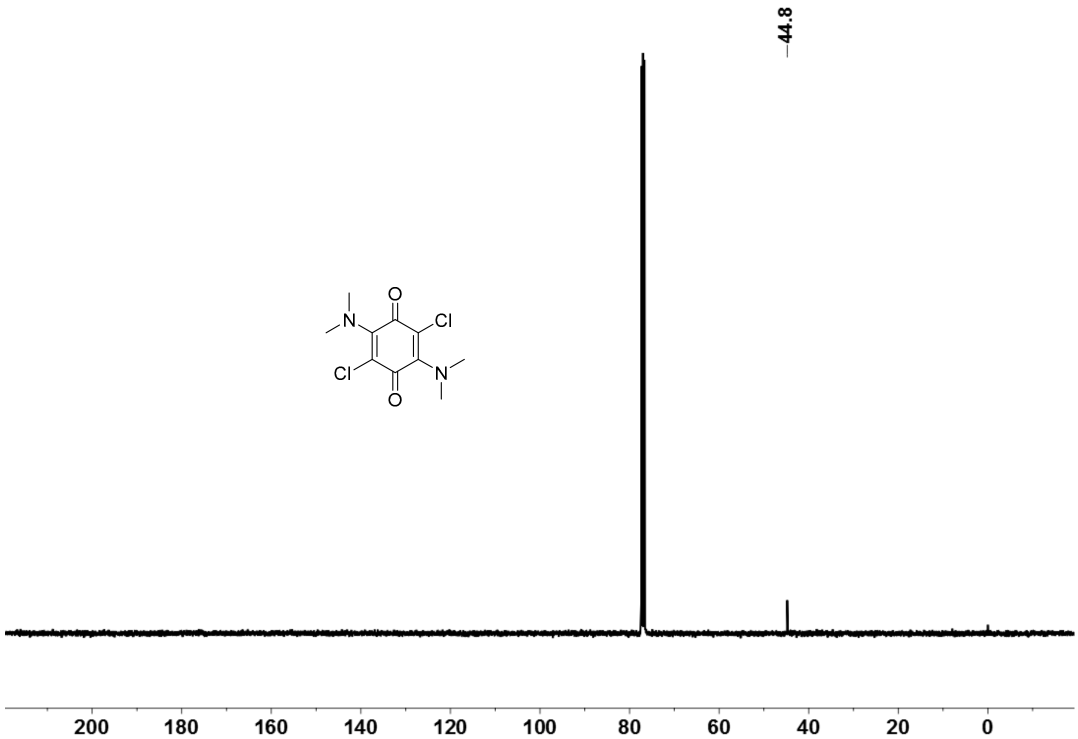

Figure S12.^{13}C NMR spectrum (101 MHz, $CDCl_3$, 298K) of 2,5-dichloro-3,6-bis(dimethylamino)cyclohexa-2,5-diene-1,4-dione

UV-vis spectral of 1 and DDQ Photos of TCBQ before and after exposure to different vapor amines.

2. Photos of TCBQ before and after exposure to different vapor amines.

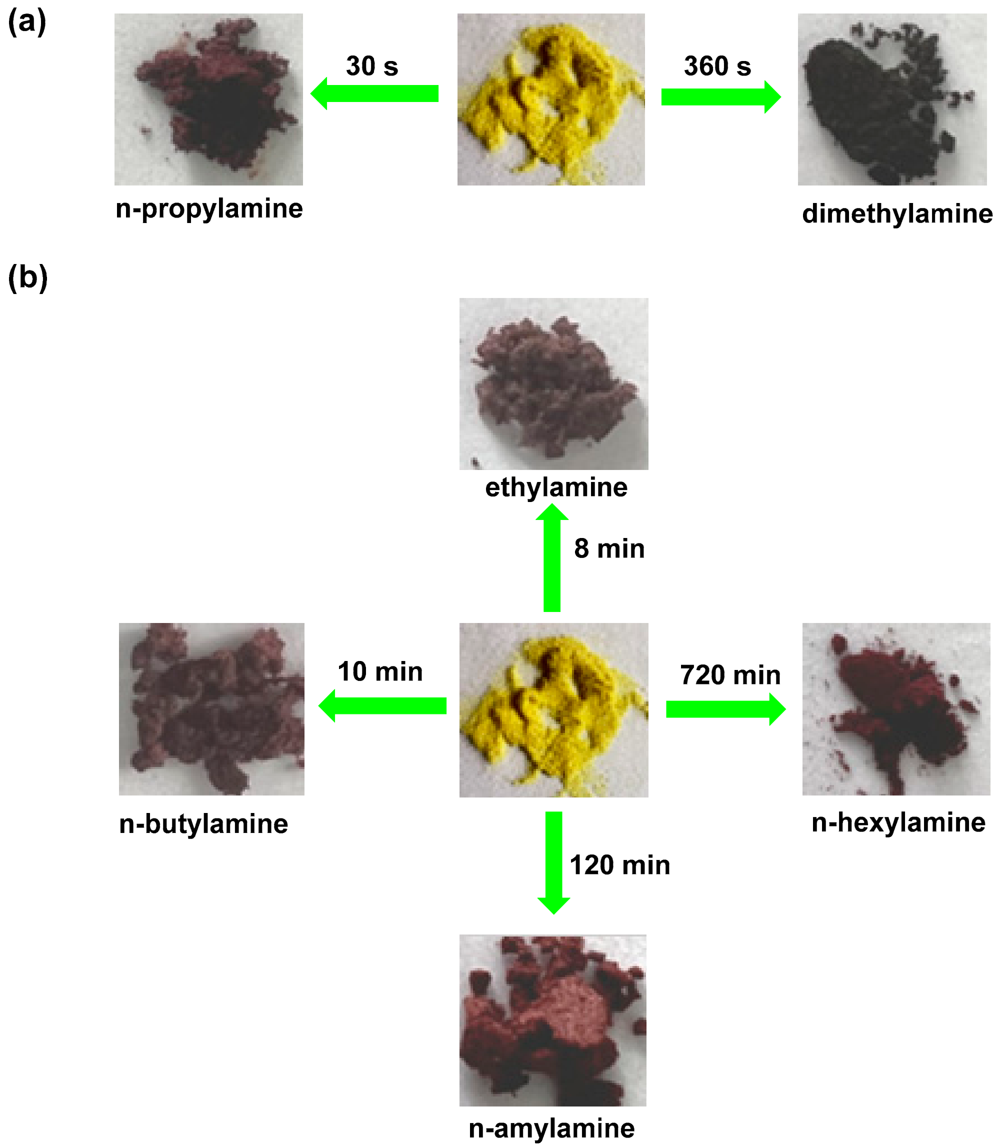

Figure S13. Photos of TCBQ before and after exposure to different vapor amines at different times.

3. Crystal data

Crystal data and structure refinement for TCBQ+C2.

Identification code	TCBQ+C2
Empirical formula	$C_{10}H_{12}Cl_2N_2O_2$
Formula weight	263.12
Temperature/K	296.15
Crystal system	triclinic
Space group	P-1
a/Å	4.752(4)
b/Å	7.553(6)
c/Å	8.500(6)
α/°	99.595(9)
β/°	92.056(9)
γ/°	105.923(9)
Volume/Å^3	288.2(4)
Z	1
ρ_{calc}g/cm^3	1.516
μ/mm^{-1}	0.549
F(000)	136.0
Crystal size/mm^3	0.2 × 0.1 × 0.1
Radiation	MoKα (λ = 0.71073)
2Θ range for data collection/°	4.878 to 54.988
Index ranges	$-6 \leq h \leq 6$, $-9 \leq k \leq 9$, $-10 \leq l \leq 10$
Reflections collected	3207
Independent reflections	1271 [R_{int} = 0.0219, R_{sigma} = 0.0315]
Data/restraints/parameters	1271/0/97
Goodness-of-fit on F^2	1.068
Final R indexes [I>=2σ (I)]	R_1 = 0.0390, wR_2 = 0.0926
Final R indexes [all data]	R_1 = 0.0530, wR_2 = 0.0987
Largest diff. peak/hole / e Å^{-3}	0.33/-0.19

Crystal data and structure refinement for TCBQ+C3.

Identification code	TCBQ+C3
Empirical formula	$C_{12}H_{16}Cl_2N_2O_2$
Formula weight	291.17
Temperature/K	296.15
Crystal system	triclinic
Space group	P-1
a/Å	4.765(3)
b/Å	7.470(4)
c/Å	10.096(5)
α/°	77.250(5)
β/°	78.671(6)
γ/°	86.462(6)
Volume/Å^3	343.6(3)
Z	1
ρ_{calc}g/cm^3	1.407
μ/mm^{-1}	0.468
F(000)	152.0
Crystal size/mm^3	0.2 × 0.2 × 0.1
Radiation	MoKα (λ = 0.71073)
2Θ range for data collection/°	4.212 to 54.98
Index ranges	$-6 \le h \le 6$, $-9 \le k \le 9$, $-12 \le l \le 13$
Reflections collected	3814
Independent reflections	1522 [R_{int} = 0.0149, R_{sigma} = 0.0185]
Data/restraints/parameters	1522/0/114
Goodness-of-fit on F^2	1.029
Final R indexes [I>=2σ (I)]	R_1 = 0.0394, wR_2 = 0.1055
Final R indexes [all data]	R_1 = 0.0490, wR_2 = 0.1123
Largest diff. peak/hole / e Å^{-3}	0.39/-0.14

Crystal data and structure refinement for TCBQ+C4.

Identification code	TCBQ+C4
Empirical formula	$C_{14}H_{20}Cl_2N_2O_2$
Formula weight	319.22
Temperature/K	296.15
Crystal system	monoclinic
Space group	$P2_1/n$
a/Å	7.426(6)
b/Å	4.668(4)
c/Å	22.114(19)
α/°	90
β/°	93.448(11)
γ/°	90
Volume/Å^3	765.1(12)
Z	2
ρ_{calc}g/cm^3	1.386
μ/mm^{-1}	0.427
F(000)	336.0
Crystal size/mm^3	0.45 × 0.42 × 0.21
Radiation	MoKα (λ = 0.71073)
2Θ range for data collection/°	3.69 to 49.956
Index ranges	$-8 \leq h \leq 8$, $-5 \leq k \leq 5$, $-26 \leq l \leq 26$
Reflections collected	5100
Independent reflections	1302 [R_{int} = 0.0788, R_{sigma} = 0.0763]
Data/restraints/parameters	1302/0/92
Goodness-of-fit on F^2	1.157
Final R indexes [I>=2σ (I)]	R_1 = 0.0788, wR_2 = 0.1501
Final R indexes [all data]	R_1 = 0.1273, wR_2 = 0.1639
Largest diff. peak/hole / e Å^{-3}	0.34/-0.25

Crystal data and structure refinement for TCBQ+C5.

Identification code	TCBQ+C5
Empirical formula	$C_{16}H_{24}Cl_2N_2O_2$
Formula weight	347.27
Temperature/K	296.15
Crystal system	triclinic
Space group	P-1
a/Å	4.7504(16)
b/Å	7.487(3)
c/Å	12.737(4)
α/°	104.371(3)
β/°	97.697(4)
γ/°	91.279(4)
Volume/Å^3	434.2(3)
Z	1
ρ_{calc}g/cm^3	1.328
μ/mm^{-1}	0.382
F(000)	184.0
Crystal size/mm^3	0.2 × 0.2 × 0.1
Radiation	MoKα (λ = 0.71073)
2Θ range for data collection/°	3.336 to 54.994
Index ranges	-6 ≤ h ≤ 6, -9 ≤ k ≤ 9, -16 ≤ l ≤ 16
Reflections collected	4789
Independent reflections	1930 [R_{int} = 0.0175, R_{sigma} = 0.0234]
Data/restraints/parameters	1930/0/148
Goodness-of-fit on F^2	1.069
Final R indexes [I>=2σ (I)]	R_1 = 0.0373, wR_2 = 0.0948
Final R indexes [all data]	R_1 = 0.0506, wR_2 = 0.1002
Largest diff. peak/hole / e Å^{-3}	0.20/-0.17

Crystal data and structure refinement for TCBQ+C6.

Identification code	TCBQ+C6
Empirical formula	$C_9H_{14}ClNO$
Formula weight	187.66
Temperature/K	296.15
Crystal system	triclinic
Space group	P-1
a/Å	4.722(15)
b/Å	7.40(2)
c/Å	14.97(5)
α/°	91.92(4)
β/°	97.45(3)
γ/°	107.18(3)
Volume/Å^3	494(3)
Z	2
ρ_{calc}g/cm^3	1.262
μ/mm^{-1}	0.341
F(000)	200.0
Crystal size/mm^3	0.2 × 0.2 × 0.1
Radiation	MoKα (λ = 0.71073)
2Θ range for data collection/°	2.752 to 49.998
Index ranges	-5 ≤ h ≤ 5, -8 ≤ k ≤ 8, -17 ≤ l ≤ 17
Reflections collected	3331
Independent reflections	1712 [R_{int} = 0.0259, R_{sigma} = 0.0457]
Data/restraints/parameters	1712/0/110
Goodness-of-fit on F^2	1.140
Final R indexes [I>=2σ (I)]	R_1 = 0.0780, wR_2 = 0.2221
Final R indexes [all data]	R_1 = 0.0975, wR_2 = 0.2332
Largest diff. peak/hole / e Å^{-3}	0.38/-0.27

4. Videos of TCBQ color change caused by n-propylamine

Solid of TCBQ

solid.mp4

Test paper prepared by TCBQ

test paper.mp4
